# Effects of transcutaneous electrical nerve stimulation (TENS) and interferential currents (IFC) in patients with nonspecific chronic low back pain: randomized clinical trial

**DOI:** 10.1590/S1516-31802011000400003

**Published:** 2011-05-05

**Authors:** Ligia Maria Facci, Jean Paulus Nowotny, Fabio Tormem, Virgínia Fernandes Moça Trevisani

**Affiliations:** 1 MD. Physiotherapist, Centro Universitário de Maringá (CESUMAR), Maringá, Paraná, Brazil.; 2 MD, PhD. Rheumatologist, Universidade Federal de São Paulo – Escola Paulista de Medicina (Unifesp- EPM), São Paulo, Brazil.

**Keywords:** Physical therapy (specialty), Rehabilitation, Electric stimulation therapy, Back pain, Spine, Fisioterapia (especialidade), Reabilitação, Terapia por estimulação elétrica, Dor nas costas, Coluna vertebral

## Abstract

**CONTEXT AND OBJECTIVE:**

Transcutaneous electrical nerve stimulation (TENS) and interferential current are the most used electrotherapy methods, although there is little scientific evidence to support their use. The aim of this study was to compare the effects of TENS and interferential current among patients with nonspecific chronic low back pain.

**DESIGN AND SETTING:**

Single-blind randomized controlled trial in the Department of Physiotherapy, Centro Universitário de Maringá.

**METHODS:**

One hundred and fifty patients were randomly divided into three groups: TENS (group 1), interferential current (group 2) and controls (group 3). The patients designated for electrotherapy received ten 30-minute sessions, while the control group remained untreated. All patients and controls were evaluated before and after treatment using a visual analog scale and the McGill Pain and Roland Morris questionnaires, and regarding their use of additional medications.

**RESULTS:**

There was a mean reduction on the visual analog scale of 39.18 mm with TENS, 44.86 mm with interferential current and 8.53 mm among the controls. In the Roland Morris questionnaire, group 1 had a mean reduction of 6.59; group 2, 7.20; and group 3, 0.70 points. In group 1, 84% of the patients stopped using medications after the treatment; in group 2, 75%; and in group 3, 34%. There was no statistically significant difference between the TENS and interferential current groups (P > 0.05); a difference was only found between these groups and the controls (P < 0.0001).

**CONCLUSION:**

There was no difference between TENS and interferential current for chronic low back pain treatment.

**CLINICAL TRIAL REGISTRATION:**

NCT01017913.

## RESUMO

**CONTEXTO E OBJETIVO:**

Estimulação elétrica nervosa transcutânea (TENS) e corrente interferencial são os métodos de eletroterapia mais utilizados, embora haja poucas evidências científicas que suportem seu uso. O objetivo deste estudo foi comparar os efeitos da TENS e da corrente interferencial em pacientes com lombalgia crônica não específica.

**TIPO DE ESTUDO E LOCAL:**

Ensaio clínico randomizado, simples-cego, no Departamento de Fisioterapia do Centro Universitário de Maringá.

**MÉTODOS:**

Cento e cinquenta pacientes foram randomicamente divididos em três grupos: TENS (grupo 1), corrente interferencial (grupo 2) e controle (grupo 3). Os pacientes designados à eletroterapia receberam 10 sessões de 30 minutos, enquanto o grupo controle permaneceu sem tratamento. Todos os pacientes e os controles foram avaliados antes e depois do tratamento usando a escala visual analógica, os questionários McGill de dor e Roland Morris, e quanto ao uso de medicamentos.

**RESULTADOS:**

Houve redução média na escala visual analógica de 39,18 mm com TENS, de 44,86 mm com a corrente interferencial e 8.53 mm no grupo controle. No questionário Roland Morris, o grupo 1 teve redução média de 6,59, o grupo 2 de 7,20 e o grupo 3 de 0,70 pontos. 84% dos pacientes do primeiro grupo, 75% no segundo e 34% no terceiro grupo cessaram a medicação depois do tratamento. Não foi encontrada diferença estatisticamente quando comparados os grupos de TENS e corrente interferencial (P > 0,05), apenas quando comparados estes grupos com o controle (P < 0,0001).

**CONCLUSÕES:**

Não há diferença entre TENS e corrente interferencial no tratamento de pacientes com lombalgia crônica.

## INTRODUCTION

Complaints of low back pain with or without irradiation are the second most common reason why workers seek healthcare assistance.^1^ The main aims of low back pain treatment are to reduce the pain and improve functional capability, while bearing in mind that these effects only occur through exercises that are linked to other resources.^2,3^ In some patients, however, the pain induces significant limitations on physical capability and impedes exercising. If the pain is under control, patients will be more capable of carrying out the program of activities. This provides justification for using electrotherapy.^4,5^

Electrotherapy, which is a noninvasive, non-pharmacological method involving transcutaneous electrical stimulation, is an additional alternative for low back pain management. The electrotherapy methods most used in clinical practice are transcutaneous electrical nerve stimulation (TENS) and interferential currents (IFC).^6^

Many researchers have investigated the effectiveness of TENS for treating chronic low back pain.^7-11^ However, most studies have not found statistically meaningful results, in comparison with placebo groups.^11^

Some studies on IFC application have been performed, to investigate its effects on induced pain^12,13^ and in relation to different diseases.^14-16^ Nonetheless, in relation to application for low back pain, there is lack of investigations^17-21^ and its effects remain unexplained.

Recently, some studies were conducted to compare the analgesic effects of TENS and IFC among healthy individuals with induced pain. No meaningful differences between them were found.^13^ These studies advocated continual use of electric currents for pain relief. However, we were unable to find any studies comparing these two techniques for treating chronic low back pain.

## OBJECTIVE

The purpose of the present study was to compare the analgesic effects of TENS and IFC among nonspecific chronic low back pain patients.

## METHODS

### Participants

The subjects for this study were recruited from a waiting list at Cesumar (Centro Universitário de Maringá), in the city of Maringá, State of Paraná, Brazil. To be included in this study, they had to be more than 18 years old and had to have been seeking treatment (after assessment by a doctor) for chronic low back pain, defined as pain localized below the scapulae and above the cleft of the buttocks, with or without leg pain, which they had had for more than three months. Their pain had to be nonspecific, meaning that no specific cause was detectable, such as infection, neoplasms, metastasis, osteoporosis, rheumatoid arthritis, fractures or inflammatory processes. Ethical approval for this experiment was obtained from the Ethics Committees of Unifesp (Universidade Federal de São Paulo) and Cesumar.

The following patients were excluded from the study: individuals who had had low back pain for less than three months; individuals who were receiving treatment for their pain with another method at the same time, except for medicines; pregnant women; patients who had undergone vertebral column surgery (less than three months before the time of this study); individuals with contraindications against electrotherapy, such as skin lesions, abnormal sensitivity, infectious and blood diseases, heart pacemakers or inability to answer questionnaires; patients with fibromyalgia; individuals with psychiatric problems; and individuals who refused to participate or were unwilling to follow a protocol lasting for two weeks.

### Procedures

After selection through consultation with a doctor, the patients provided their written consent and were given an opportunity to ask any questions regarding the procedure. The patients were examined by an independent physiotherapist, who used a pre-prepared card composed of several instruments: visual analog scale (VAS),^22^ Brazilian version of the McGill Pain Questionnaire classified according to the number of words chosen (NWC), Pain Rating Index (PRI), Pain Intensity Index (PPI)^23-25^ and Roland-Morris Disability Questionnaire (RMDQ).^26^ The examination was done by an independent physiotherapist before and after the protocol of ten treatment sessions. This examiner did not follow the treatment and did not know which group the patients had been included in. After each treatment session, however, the pain intensity was also evaluated using VAS among the patients that received TENS and interferential current.

After evaluation by the physiotherapist, the patients were randomized, through numbers created by a computer, into three groups: 1) TENS (n = 50); 2) interferential current (n = 50); 3) controls (n = 50). The randomized design was balanced in groups of 50. A set of sealed, sequentially numbered opaque envelopes was used for study group assignment. Thus, the study was single-blinded, i.e. the examiner had no contact with the patient during the treatment, and the patient was instructed not to report what assistance had been received during the sessions.

### Intervention

Two types of equipment were used: Endophasys I-ET9702 (interferential current) and TENYS-ET 9771 (TENS) C (KLD Equipment, Amparo, São Paulo). The treatment was applied over a two-week period, in ten sessions. For both intervention groups, the stimulation was administered for 30 minutes, using a strong, but comfortable intensity that was adjusted according to each patient's sensitivity. Four self-adhesive Valutrode electrodes with dimensions of 5 × 5 cm were placed over the T_12_ and S_1_ lines.

The TENS equipment was calibrated at a frequency of 20 Hz and a pulse width of 330 ms, with two channels. The IFC was adjusted to a base frequency of 4000 Hz, with a modulation frequency range of 20 Hz, ΔF of 10 Hz and slope of 1/1, in quadripolar mode. The frequency of 20 Hz was chosen in accordance with suggestions from previous study results.^27-30^

All the patients received guidance folders about vertebral column care. This was the only intervention administered to the patients who were chosen for the control group. These patients remained on a waiting list for 15 days, until beginning conventional physiotherapy treatment at the same clinic.

The VAS was applied every day, before and after the session. In addition, the patients filled out a questionnaire in which, aided by a physiotherapist, they stated for how long their pain relief after the session had lasted and whether they had used any painkillers or anti-inflammatory drugs prescribed by the doctor, and what dosages they used.

After completing the 10 sessions, the patients were reassessed by an independent evaluator who used the same instruments. In the event of dropout, i.e. patients not returning for reassessment, they were asked why they had given up the treatment and what medications they had used, when these dropout patients could be located.

### Statistical analysis

All data were analyzed using Statistica version 7 and SAS version 9.1.^31^ Baseline characteristics were compared using the Shapiro-Wilks test for continuous variables and then analysis of variance (Anova) for measuring independent data. The characteristics of the patients who finished the treatment were compared with those of the lost patients, using one-way Anova and the Kruskal-Wallis test.

The decreases in VAS and RMDQ scores were calculated by subtracting the end value from the initial value, expressed as a percentage. Anova for repeated measurements was used to determine the effects among the treatment groups by comparing their mean values. To compare frequencies between groups, Student's t test was used with a significance level of 5%. The decreases in VAS in groups 1 and 2 following each session and the PPI, NWC and PRI indexes before and after the treatment in groups 1, 2 and 3 were investigated using Anova for repeated measurements, and the means for the groups were also compared using Duncan's test.

For the pain intensity variable alone, examined using VAS, the statistical analysis was performed with all the patients selected at random according to intention to treat. For this, all the patients included in the study were taken into consideration. For this purpose, the reasons for giving up the treatment were investigated. The patients were classified as with pain or without pain, independent of their pain intensity. The signal hypothesis test and Wilcoxon test were then applied to investigate whether there were any changes in pain levels in each patient group.

The consumption of medications was analyzed by means of simple frequency tables. The McNemar test was applied to investigate whether there was any association between the use of medicine and the treatment. The use of non-steroidal anti-inflammatory drugs and analgesic drugs was analyzed by means of simple frequency tables, with double data entry.

### Sample size calculation

The type I error was taken to be α = 0.05 and the type II error was taken to be β = 0.10, with a confidence interval of 95%, assuming a 90% likelihood of demonstrating a 30% difference in the TENS group. From this, improvements of 10% in interferential current^18^ and 37% in TENS^10^ had previously been observed. To achieve this, the sample size was set at 47 patients, supposing a total of 150 participants.^27^ The same method was used to investigate the difference between the TENS and control groups, supposing a 90% likelihood of 33% improvement in the TENS group, since a previous study had observed a 4% improvement in the control group and 37% in the TENS group.^10^ To achieve this, the sample size was set at 26 patients for each group, with a total of 78 participants.

## RESULTS

**Figure 1.**
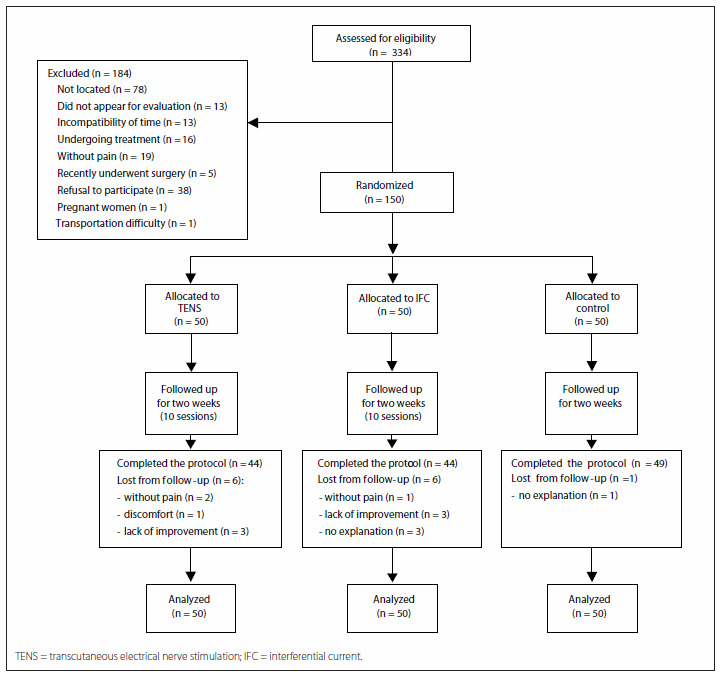
Patient distribution for the study.

The total number of low back pain patients selected was 334. Out of these, 184 were excluded for a variety of reasons, and thus 150 patients were included in the study and evaluated. Among these, fifty were allocated by randomization to group 1 (TENS), fifty to group 2 (IFC) and fifty to group 3 (controls). Thirteen patients (8.66%) gave up during the treatment, of whom six (12%) were in group 3 (2%). Thus, 137 patients completed the treatment protocol (Figure 1).

Table 1 shows the demographic features of the population included in this study according to group allocation. Only the initial pain intensity differed significantly between any of the groups (only between groups 1 and 3) (P = 0.0086), at a significance level of 5%.

**Table 1.** Patient characteristics at baseline

Category	Groups			P-value
	1	2	3	
Age (years)				
Mean	49.63 ± 15.52	45.32 ± 17.05	46.56 ± 15.197	0.38
CI	(45; 54.03)	(40.47; 50.17)	(42.24; 50.88)	
Sex (%)				
Female	70.0	74.0	74.0	0.87
Male	30.0	26.0	26.0	
Height (cm)				
Mean	1.61 ± 0.08	1.62 ± 0.09	1.63 ± 0.08	0.51
CI	(1.59; 1.63)	(1.60; 1.65)	(1.61; 1.65)	
Weight (kg)				
Mean	70.41 ± 12.30	69.88 ± 14.93	67.93 ± 12.40	0.61
CI	(66.91; 73.90)	(65.64; 74.13)	(64.41; 71.46)	
Body mass index				
Mean	27.1 ± 4.74	26.64 ± 5.96	25.50 ± 3.62	0.24
CI	(25.75; 28.45)	(24.95; 28.33)	(24.47; 26.53)	
Marital status (%)				
Single	18.0	22.0	24.0	
Married	70.0	60.0	68.0	0.30
Widowed	6.0	2.0	4.0	
Divorced	6.0	16.0	4.0	
Ethnicity (%)				
White	88.0	88.0	92.0	0.75
Black	12.0	12.0	8.0	
History of low back pain (%)				
3 --| 6 months	16.0	14.0	20.0	
6 --| 12 months	18.0	16.0	14.0	0.80
More than 12 months	66.0	70.0	66.0	
Pain distribution (%)				
Low back pain	78.0	78.0	70.0	0.36
Low back pain and sciatica	22.0	22.0	30.0	
Physical activity practiced (%)				
Yes	20.0	20.0	18.0	0.95
No	80.0	80.0	82.0	
Initial pain intensity (VAS) (mm)				
Mean	46.5 ± 28.6	56.6 ± 24.9	69.4 ± 25.6	< 0.01
CI	(38.4; 54.7)	(49.6; 63.7)	(55.7; 70.2)	
Initial functional disability score (RMDQ)				
Mean	13.36 ± 5.41	14.22 ± 4.79	15.41 ± 5.45	0.15
CI	(11.82; 14.9)	(12.86; 15.58)	(13.84; 16.97)	
Initial use of medications (%)				
Yes	64.6	69.0	66.6	0.97
No	35.4	31.0	33.4	

* RMDQ = Roland-Morris questionnaire; VAS = visual analog scale; CI = 95% confidence interval.

Independent of group allocation, comparison of patient characteristics between individuals who finished the treatment protocol (n = 137) and those who did not (n = 13) using one-way Anova and the Kruskal-Wallis test showed that there was no statistically significant difference between these groups (Table 2).

**Table 2.** Patient characteristics at baseline: comparison between patients who finished the treatment and patients lost from follow-up

Category	Finished	Lost from follow-up	*x* ^2^	P-value
n		137			13
Age (years)				
Mean	47.63 ± 16.06	42.31 ± 14.22		
CI	(44.91; 50.34)	(33.71; 50.90)	1.2827	0.25
Sex				
Female	72.99	69.23	0.0840	0.77
Male	27.01	30.77		
Height (cm)				
Mean	1.62 ± 0.09	1.62 ± 0.07		
CI	(1.61; 1.63)	(1.8; 1.67)	0.0953	0.75
Weight (kg)				
Mean	69.86 ± 13.51	64.59 ± 8.56		
CI	(67.58; 72.15)	(59.41;69.77)	1.0129	0.31
Body mass index				
Mean	26.58 ± 4.91	24.62 ± 4.35		
CI	(25.75;27.41)	(21.99;27.24)	1.3424	0.24
Marital status (%)				
Single	20.44	30.77		
Married	67.15	53.85		
Widowed	3.65	7.69	0.2522	0.61
Divorced	8.76	7.69		
Ethnicity (%)				
White	90.51	76.92	22852	013
Black	9.49	23.08		
History of low back pain (%)				
3 --| 6 months	16.79	15.38		
6 --| 12 months	16.79	7.69	0.4373	0.50
More than 12 months	66.42	76.92		
Initial pain intensity (VAS)				
Mean	55.2 ± 27.2	56.6 ± 26.5		
CI	(50.6; 59.8)	(40.6; 72.7)	0.0036	0.95
Initial functional disability score (RMDQ)				
Mean	14.38 ± 5.27	13.69 ± 5.23	0.2521	0.61
CI	(13.49; 15.28)	(10.53;16.86)		
Physical activity practiced (%)				
Yes	19.71	15.38		
No	80.29	84.62	0.1414	0.70
Initial use of medications (%)				
Yes	64.96	61.54	00606	080
No	35.04	38.46		

* RMDQ = Roland-Morris Disability Questionnaire; VAS = visual analog scale; CI = 95% confidence interval.

## Outcomes evaluated

### Pain intensity (VAS)

**Figure 2.**
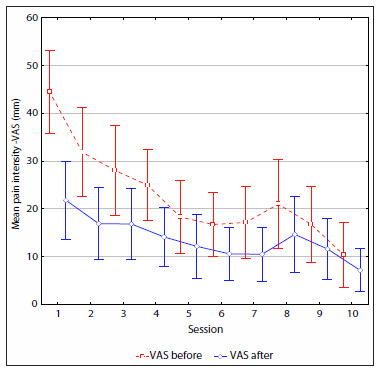
Pain intensity (VAS) at the beginning and at the end of each session for group 1 patients.

**Figure 3.**
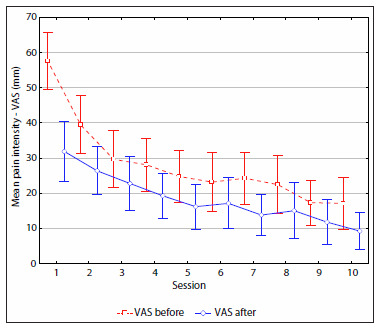
Pain intensity (VAS) at the beginning and at the end of each session for group 2 patients.

Figures 2 and 3 shows that there was a trend for the mean VAS to decrease over the course of the sessions, in both group 1 (TENS) and group 2 (IFC). There was no difference in mean pain intensity between the groups before each treatment session (P = 0.19), since the group means for the sessions were the same (P < 0.05). However, there was a difference between sessions (P < 0.01) only for the first session of group 1 (4.44) compared with group 2 (5.75). Thus, it could be seen that although the pain intensity was heterogeneous at the beginning of the study, it became homogeneous between the groups from the second session onwards.

The mean pain decreased over the course of the treatment in both groups. Group 2 presented a more significant reduction in the seventh session (Figures 2 and 3). Anova for repeated measurements did not show any difference between the session means (P < 0.01) or between the group means (P = 0.60), since there was no interaction between groups and sessions (P = 0.55).

**Figure 4.**
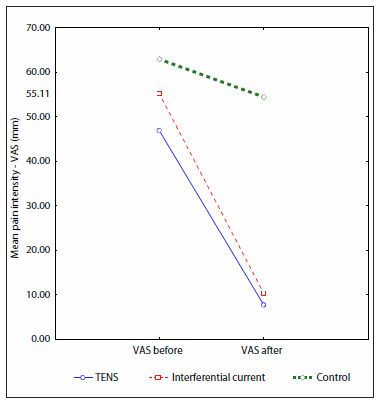
Initial and final mean pain intensity (VAS) according to treatment group.

Assessment of mean pain intensity (VAS) before and after the treatment showed that the means decreased in all three groups. The IFC group presented a mean decrease in VAS of 4.48 cm; the TENS group, 3.91 cm; and the control group, 0.85 cm (Figure 4). Although there was no statistically significant difference between groups 1 and 2 using Duncan's test, the IFC patents presented greater pain reduction. The control group presented statistically significant differences with the other two groups.

The patient distribution of changes in pain intensity after the treatment showed that 84% of group 1, 82% of group 2 and 56% of group 3 had pain reductions. There were statistically significant differences between groups 1 and 3 (P < 0.01) and between groups 2 and 3 (P < 0.01). Setbacks (increases in pain) occurred in 4% of groups 1 and 2, and in 38% of group 3.

The intention-to-treat analysis included all the patients with VAS pain evaluations, and the worst results were considered to be losses. The results from this evaluation were statistically significant in relation to decreased pain intensity in groups 1 and 2, but not in group 3 (Table 3).

**Table 3.** Pain intensity (visual analog scale, VAS) before treatment, in intention-to-treat analysis groups

Pain intensity	Group 1	Group 2	Group 3
	Frequency (%)	Frequency (%)	Frequency (%)
Without pain	31 (62%)	28 (56%)	1 (2%)
With pain	19 (38%)	22 (44%)	49 (98%)
Total	50 (100%)	50 (100%)	50 (100%)

### Pain evaluation using McGill Pain Questionnaire (MPQ)

The pain analysis using the McGill Pain Questionnaire (MPQ) showed that for the PPI, PRI and NWC indices before the treatment, the means for groups 1 and 3 were different, but groups 1 and 3 were each the same as group 2 (P < 0.05). After the treatment, the means for groups 1 and 2 were different from the mean for group 3 but were the same as each other. The decrease in PPI was greater in group 1 (-1.45) and the decreases in PRI (-25.34) and NWC (8.29) were greater in group 2. These decreases in PPI, PRI and NWC were evaluated using Anova, which showed differences in the decrease between the groups: PPI (P < 0.01), PRI (P < 0.01) and NWC (P < 0.01). By applying Duncan's test (post-hoc test), group 3 was found to present differences with the other groups, in relation to PPI, PRI and NWC. Groups 1 and 2 were similar to each other in relation to PPI and NWC, but were different in relation to PRI (Table 4).

**Table 4.** Pain intensity index, pain rating index and mean number of words chosen among the groups.

Group	Pain intensity index		Pain rating index		Number of words chosen	
	Before	After	Before	After	Before	After
1	1.95^a^	0.50^a^	25.63^a^	7.97^a^	11.02^a^	4.22^a^
2	2.22^ab^	0.81^a^	35.11^b^	9.77^a^	13.25^b^	4.95^a^
3	2.53^b^	1.87^b^	35.08^b^	31.55^b^	13.97^b^	13.85^b^

Note:

Means with different superscript letters are statistically different according to Duncan's test and Student's t test at a significance level of 5%.

### Duration of analgesia

**Figure 5.**
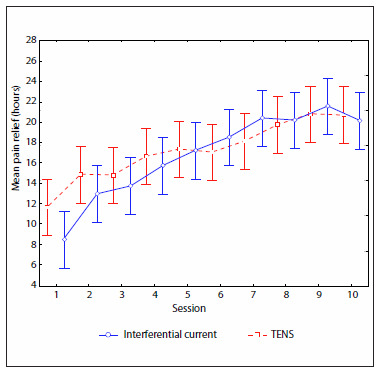
Comparison of mean pain relief in transcutaneous electrical nerve stimulation (TENS) and interferential current groups.

The duration of the analgesia caused by TENS and IFC in each session was measured in hours, at 24-hour intervals after the patient finished the session (Figure 5). There were no statistically significant differences between the group values (P = 0.77), since there were no interactions between groups and sessions (P = 0.54), but the differences between the sessions were significant (P < 0.01). There was an increasing trend in the mean duration of analgesia over the course of the sessions, up to a climax in the tenth session for the TENS group and the ninth for the IFC.

### Disability

**Figure 6.**
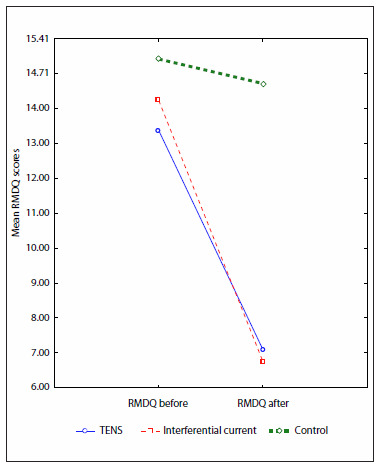
Changes in Roland-Morris Disability Questionnaire (RMDQ) scores from before to after treatment, in the TENS, interferential current and control groups.

Disability was analyzed using the RMDQ, and showed improvements in all the groups. At the beginning of the study, the three groups were homogeneous (P = 0.15), but after the treatment, a difference between group 3 and the other two groups was found (P < 0.01). The decrease in RMDQ score was significant in groups 1 and 2 (Figure 6), and for the three groups (P < 0.01).

### Use of medications

There were reductions in the consumption of non-steroidal anti-inflammatory drugs (NSAIDs) and analgesic drugs, such that 84% of the patients in group 1, 75% in group 2 and 34% in group 3 stopped using these medications after the treatment. The statistical analysis showed that the proportions of the patients who stopped using the medications were similar between groups 1 and 2, but different from group 3.

Frequency evaluations relating to the use of analgesics and NSAIDs in the three groups during the treatment showed that NSAID use was bigger than analgesic use. There were no differences in the numbers of patients in group 3 who were using between one and ten analgesics and those using NSAIDs. We emphasize that in all the groups, most of the patients were not using any drugs. Group 2 presented the largest number of patients (91.3%) who were not using drugs. Group 3 was bigger than the others (28.26%) in relation to using between one and ten analgesics and more than ten NSAIDs.

## DISCUSSION

According to the results presented, TENS and IFC produced significant effects in relation to pain intensity reduction, disability improvement and reduction in medication consumption. These results did not occur in the control group. Although 13 patients (8.66%) did not finish the study, this small number of losses was not enough to influence the significant results of this clinical trial.

It is difficult to achieve complete masking of patients in physiotherapy investigations, since there are differences between visual sensory and alternative treatments.^28^ We chose not to apply switched-off electrical equipment to the control group in this study, given that all three groups investigated received guidance relating to ergonomic spinal care.

Some previous studies observed the effects of TENS in relation to IFC in situations of experimentally induced pain.^29-36^ Johnson et al.^29^ and Cramp et al.^30^ did not find significant differences in pain intensity among healthy people subjected to both types of electric current. Similar results were found by Johnson and Tabasam^33^ and Cheing and Hui-Chan,^35^ with IFC showing a slight advantage over TENS in some ways. Although these previous results were not similar to those of the present study, it was not possible to make direct comparisons between the previous and present results because of the different methodologies used in the research.

To investigate TENS in low back pain patients, Melzack et al.^7^ carried out a randomized clinical trial comparing its effects with the effects from massage, in cases of chronic or acute low back pain. From the McGill Pain Questionnaire (MPQ), the TENS group was found to present a reduction in PRI of 69.5%, a reduction in PPI of 80.8%, a range of movement improvement and a reduction in pain intensity (VAS) of 84%. The effectiveness of the interventions, as determined by the patients’ percentage improvement, was 38% in the massage group and 85% in the TENS group. There was no statistically significant difference between the groups.

Marchand et al.^9^ randomized 48 patients with chronic low back pain into three groups (TENS, placebo and control). Comparing TENS and placebo, they found a 43% reduction in pain intensity in the TENS group and 17% in the placebo group. However, the methodology of their study can be criticized because it included patients with different diseases, such as ankylosing spondylitis and rheumatoid arthritis, thus differing from the present study, which did not use placebo for comparisons of possible results.

In a randomized clinical trial, Deyo et al.^8^ compared the effectiveness of TENS and a stretching program and did not find any significant differences between TENS and placebo after one month of treatment. Over the same period, the groups that performed workouts, whether or not in association with TENS, showed meaningful improvements in their painful state, or in function or pain frequency. Their findings are not in agreement with the present study, which detected significant differences between the treatment groups and the control group.

Cheing and Hui-Chan^10^ described the effects of a 60-minute TENS session on chronic clinical pain, acute experimental pain and chronic low back pain. In the group that received TENS, there were statistically significant decreases in mean values, by 28% during the application and 37% after the treatment, while in the placebo group the mean values decreased by 4%. Neither TENS nor placebo produced significant changes in experimentally induced pain. Methodological differences, especially relating to the duration of the treatment, make it difficult to correlate their data with the information from the present study.

In a recent systematic review, Khadilkar et al.^11^ only included two of the 47 clinical trials that had previously been performed to investigate the effects of TENS in cases of chronic low back pain.^8,10^ Even though the inclusion criteria were stated, the reviewers emphasized that there was a lack of a standardization system, and they did not find enough evidence to justify TENS use in cases of chronic low back pain.

The methodology of the present study sought to use the parameters suggested by the systematic review of Khadilkar et al.^11^ However, it is important to note that we did not include a placebo group to which switched-off electrical equipment was applied, and it was not possible to monitor long-term results. The suggestion from the present study is that electrotherapy should be used only for an initial period of treatment, so that other resources can be applied later on. Nonetheless, uncertainties regarding the causal factors of low back pain may mean that the analysis on the present results is not applicable to all patients in clinical practice.

Some experimental studies showing the analgesic effects of IFC on induced pain have been conducted.^12,13^ So far, only a small number have dealt with specific problems such as recurrent jaw pain,^14^ pain after knee surgery^15^ and pain due to fibromyalgia.^16^ Studies have recently been conducted on IFC application in cases of acute low back pain^19-21,36^ and chronic low back pain.^22,23^

Romani et al.^36^ used 20 minutes of IFC on acute low back pain patients. After the treatment, reductions in their pain could be observed using a handheld dynamometer. Hurley et al.^20^ found significant changes in pain intensity and functional capability. Previously, Hurley et al.^19^ had achieved significant improvements in acute low back pain intensity by means of different electrode positions. Although Romani et al.^36^ and Hurley et al.^19^ investigated patients with acute pain, their findings were in agreement with the reductions in pain intensity seen among the patients of the present study, which were also significant findings in their studies.

González Roig et al.^17^ divided 120 chronic low back pain patients into two intervention groups, in order of arrival: a group that received IFC and a control group that received surface warming. In both groups, the patients underwent twelve ten-minute sessions, together with Williams exercises. All the patients who received IFC obtained pain relief, although 35% did not have full resolution of their situation. In the control group, 20% did not obtain any pain relief and 61.4% did not have full resolution of their situation. However, those authors used methods that differed from those of the present study, thus making it difficult to compare the information, such as in relation to the infrared heating applied to the control group and the exercises in both groups.

In a randomized clinical trial, Werners et al.^18^ applied IFC to cases of chronic low back pain and compared its effect with the effect of massage, among 148 low back pain patients. Both groups underwent six ten-minute sessions, but the selection criterion of how long the patients needed to have had their complaint was not described. There were no significant differences between the groups in relation to the outcomes evaluated. IFC gave rise to a mean pain reduction of 10% immediately after the treatment and 16% after three months. Their findings were not in agreement with those of the present study, in which the mean reduction in VAS was 44.8 mm, thus emphasizing that 54% of the patients were free from pain after IFC treatment.

In a recent randomized study, Solano et al.^37^ compared 30 minutes of TENS with 30 minutes of IFC among 30 patients with acute low back pain. The TENS equipment was calibrated at a frequency of 100 Hz, with a pulse width of 150 ms, pulses of 2 Hz and four electrodes. The IFC was adjusted to a frequency modulation range of 5 Hz and spectrum of 10 Hz, with vectors. The pain reduction (mean difference) among the patients treated with IFC was 2.18 cm (31.5%) and it was 1.24 cm with TENS (18.4%). Despite the statistically meaningful results obtained, no meaningful differences were found between the groups. Both the results obtained by Solano et al.^37^ and the results from the current study emphasize that there are no differences between TENS and IFC use for low back pain patients. However, the equipment adjustments used by Solano differed from those of the present study, thus showing that the frequency values for the comparable results from TENS and interferential current were not standardized in Solano's study.

These findings show that the choice of which electric current method to use now depends on the costs of equipment acquisition and maintenance. These factors should be investigated in future studies.

Although it was decided not to implement therapeutic exercises in association with the protocol for the present study, it was found that the electrotherapy protocols even produced significant benefits relating to the patients’ functional capability. However, it must be emphasized that ongoing treatment is needed in clinical practice, with exercises, which may make the pain intensity more comfortable.

We therefore suggest that new studies should be carried out with the aim of analyzing what type of equipment is most appropriate with regard to long-term pain relief, taking into account the long-term maintenance costs and the investigation parameters of frequency regulation and pulse width.

## CONCLUSIONS

The results from this study showed that TENS and IFC had significant effects in relation to pain intensity reduction, disability improvement and reduction of medication consumption, immediately after each electrotherapy session and after ten sessions, in comparison with the controls. However, no significant differences in these resources in relation to treating patients with nonspecific chronic low back pain were observed.
